# Evaluative altmetrics: is there evidence for its application to research evaluation?

**DOI:** 10.3389/frma.2023.1188131

**Published:** 2023-07-25

**Authors:** Wenceslao Arroyo-Machado, Daniel Torres-Salinas

**Affiliations:** Department of Information and Communication Sciences, University of Granada, Granada, Spain

**Keywords:** altmetrics, social media metrics, research evaluation, evaluative altmetrics, Twitter, news, Wikipedia

## Abstract

**Introduction:**

Altmetrics have been demonstrated as a promising tool for analyzing scientific communication on social media. Nevertheless, its application for research evaluation remains underdeveloped, despite the advancement of research in the study of diverse scientific interactions.

**Methods:**

This paper develops a method for applying altmetrics in the evaluation of researchers, focusing on a case study of the Environment/Ecology ESI field publications by researchers at the University of Granada. We considered Twitter as a mirror of social attention, news outlets as media, and Wikipedia as educational, exploring mentions from these three sources and the associated actors in their respective media, contextualizing them using various metrics.

**Results:**

Our analysis evaluated different dimensions such as the type of audience, local attention, engagement generated around the mention, and the profile of the actor. Our methodology effectively provided dashboards that gave a comprehensive view of the different instances of social attention at the author level.

**Discussion:**

The use of altmetrics for research evaluation presents significant potential, as shown by our case study. While this is a novel method, our results suggest that altmetrics could provide valuable insights into the social attention that researchers garner. This can be an important tool for research evaluation, expanding our understanding beyond traditional metrics.

## 1. Introduction

The advent of social media has led to an unprecedented expansion of the spectrum of metrics used to study scientific impact and communication (Priem et al., 2012). The first firm call for the use of these metrics was made in 2010 with the Altmetrics Manifesto, which already pointed out that “broader impact metrics could also play a role in funding and promotion decisions” (Priem et al., 2010). This potential has always been in the spotlight and its realization has been viewed as the ultimate goal (Torres-Salinas et al., 2013). This revolution has led to a whole stream of research mainly focused on finding clear evidence of later citations in the early mentions of scientific publications on Twitter, Wikipedia and other social media (Costas et al., 2015). This correlation has only been found for Mendeley readers (Thelwall, 2018a), although transparency issues associated with Mendeley readership counts have hindered its study (Thelwall, 2018b).

Thus, there is an extensive altmetric landscape misaligned with traditional bibliometric indicators, which has proven useful for understanding how science is disseminated and discussed through these communication channels. Despite Twitter's well-known problem of volatility (Fang et al., 2022b), mentions made through this social network stand out due to their abundance, having the highest presence in altmetric data aggregators (Zahedi and Costas, 2018; Karmakar et al., 2021). This has given rise to a plethora of studies that analyze the coverage of scientific literature (Fang et al., 2020; Torres-Salinas et al., 2022), map its communities of interest and topics (Robinson-Garcia et al., 2018; Schalkwyk et al., 2020; Arroyo-Machado et al., 2021) or explore the different types of engagement (Fang et al., 2021, 2022a). While fewer efforts have been devoted to studying news mentions, there is no shortage of research that delves into the nature of such mentions. For example, similarities between news outlets have been mapped based on common mentions (Ortega, 2021) and the dominance of local research in the news has been studied (Yu et al., 2022). On the other hand, Wikipedia mentions, one of the earliest altmetric sources (Nielsen, 2007), have been explored through multiple perspectives, for example to analyze their citation patterns (Maggio et al., 2017) and the differences between linguistic editions (Lewoniewski et al., 2017) or to map its collaborative knowledge structure (Arroyo-Machado et al., 2020).

The evident potential of social media activity relating to science and its communication has led to the emergence of theoretical proposals for integration of evidence from different social media (Costas et al., 2020), ultimately giving rise to a new generation of social media metrics that aim to look beyond mention counts and better understand the diversity of interactions and attention around science (Díaz-Faes et al., 2019). While this new research stream is constantly evolving and growing with new proposals, no such breakthroughs have been made in the realm of research evaluation. This is no minor concern, given that the incorporation of metrics capable of reflecting social attention and knowledge transfer is currently on the political agenda (National Information Standards Organization, 2016; European Commission Directorate-General for Research Innovation et al., 2017, 2019). However, the only advances in this field are theoretical studies that rely on the empirical evidence provided by the ample altmetric literature to formulate recommendations for the use of these metrics in evaluation (Thelwall, 2018b, 2020a,b). Indeed, there is a complete absence of proposals with a practical approach exploring tangible applications for this evaluative evidence and raising awareness of the possibilities of these social metrics.

One of the main reasons for this tardy interest in the development of practical applications in the field of scientific evaluation is the lack of evidence and consensus regarding their potential as metrics of social attention.^1^ Since their inception, these metrics have been seen not only as an alternative to traditional citation-based indicators, which reflect academic impact, but also as potential indicators of social attention by capturing the interest or influence of science outside the scientific realm (Thelwall et al., 2013; Bornmann, 2014). However, the question of what constitutes social attention has raised many doubts, with the early years pointing to the possibility of a fragmented nature made up of different dimensions to which this variety of metrics can contribute (Priem et al., 2012). This has resulted in it being referred to as “unknown attention” in the absence of such concreteness and evidence (Bornmann et al., 2019). Nevertheless, these areas of attention have become increasingly delimited. For example, it is now clear that a patent citation differs from a Facebook mention in that the former reflects a commercial interest while the latter reflects a public interest (Thelwall, 2020a). Following this same premise, even applied proposals have arisen such as the InfluScience research project, which analyzes the social, media, political and educational influence of Spanish researchers through mentions on Twitter and in news outlets, policy reports and Wikipedia (InfluScience, 2022).

Therefore, if altmetrics wish to move forward they must be nourished by historical and proven proposals in the field, such as those of evaluative informetrics (Moed, 2017). This means putting an end to “bean counting” (Ràfols, 2019) and importing multidimensional and contextual practices into the altmetrics domain. To this end, it is necessary to create concrete and precise evaluative frameworks that improve the meaning and usefulness of such indicators. Some proposals to give meaning to altmetrics have been oriented toward detecting audiences, identifying local attention, measuring engagement or studying the profiles of the actors. Thus, in light of the need to apply altmetrics in real decision-making contexts and the fact that there are already sufficiently tried and tested sources (Twitter, news, Wikipedia), in which the largest volume of altmetrics mentions are usually concentrated (Torres-Salinas et al., 2023), the general objective of this paper is to present a “portfolio” of significant case studies of altmetrics and university policy decision-making context based on the principles of evaluative bibliometrics established in the latest publication by Moed (2017). Its aim is to be of use in scientific policy and decision-making processes, especially in the context of universities (Torres-Salinas et al., 2016), which are increasingly concerned with the evaluation of social attention (Bornmann et al., 2019). More specifically, the following specific objectives have been established:

- To individually explore the three altmetric sources (Twitter, news and Wikipedia) to propose practical solutions for identification of local attention and different audiences and obtain metrics that reflect engagement with mentions and characterize the profile of social actors.
- To conduct a research evaluation case study that highlights the potential of altmetrics at author level.

## 2. Methodology

As this is an exploratory study that seeks to delve into the different types of altmetric evidence for evaluation at author level, we have used a small sample of publications associated with a controlled environment, but which captures the attention of the three social media studied (Twitter, news and Wikipedia). After reviewing the InfluScience2 ranking^2^ and the altmetric ranking of Spanish researchers and institutions, the disciplines of Environmental Science and Ecology were identified as the most suitable, because in addition to high altmetric attention scores they also have a balanced presence in the three sources studied. The decision was therefore made to use as the sample publications from the Environment/Ecology ESI field published by researchers of the University of Granada (UGR), on the basis that it is one of the Spanish universities with the most experience and interest in this field.

**Figure 1:**
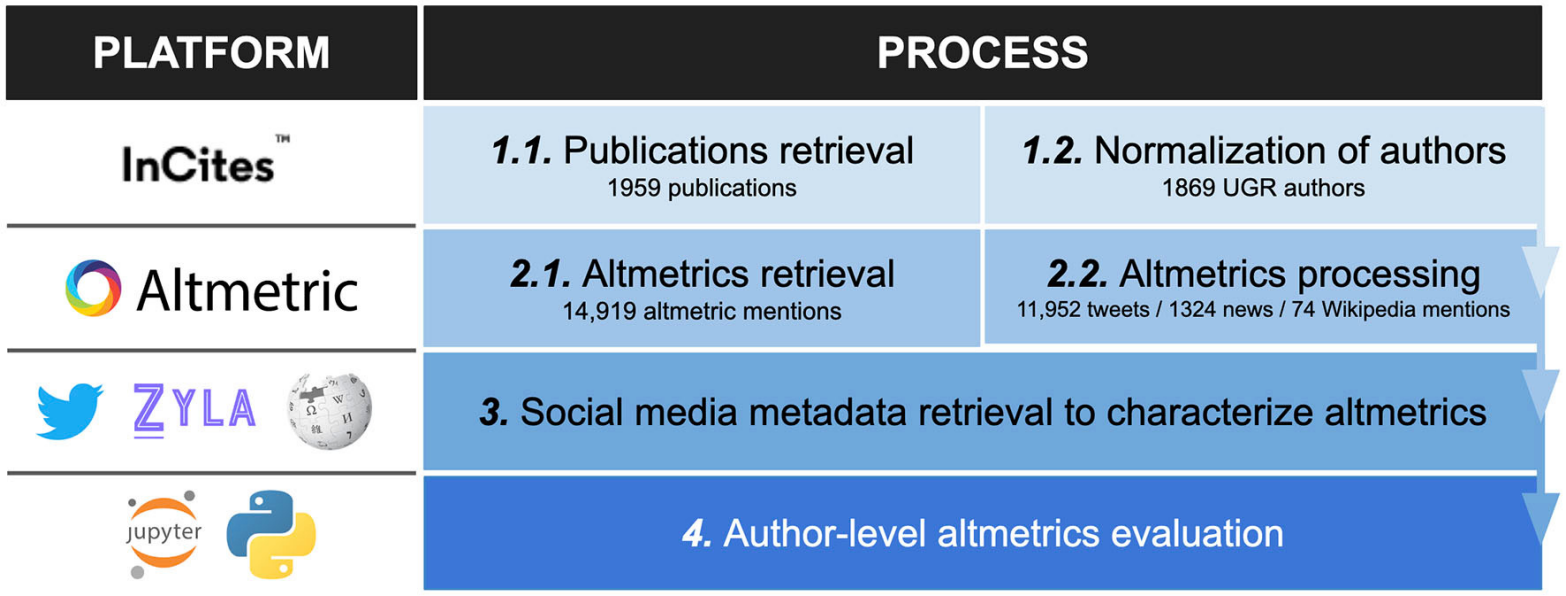
Summary of the methodological process and transformation of altmetric data for author-level evaluation.

The data retrieval process carried out on 7 February 2023 is summarized in Figure 1. Firstly, bibliographic records of the production of researchers at the University of Granada in the Environment/Ecology ESI field published between 2012 and 2021 were retrieved using InCites. A total of 1,959 publications were retrieved, of which 1,784 were articles (91%) and 135 were reviews (7%). It was not filtered according to any document type as the typologies that usually receive the most citations (articles and reviews) are not necessarily always those that also receive the most altmetric attention (Sugimoto et al., 2017). The authors were then disambiguated using the algorithm proposed by Caron and van Eck (2014), resulting in a total of 1,869 researchers affiliated with the University of Granada. Next, all the altmetric mentions of these publications from the three selected social media were retrieved by querying Altmetric.com with the DOI of the publications, identifying 1,047 publications (53%) with at least one mention in any altmetric source.^3^ Subsequently, the metadata of these mentions were complemented using additional sources such as Twitter API and Wikimedia REST API. All the Python and R scripts with data processing are available at the following GitHub repository: https://doi.org/10.5281/zenodo.7740950.

Our proposal thus focuses on the exploration of altmetric indicators selected for their use like evaluative bibliometrics, which as a whole will be capable of offering a much clearer picture of the different kinds of attention that science receives. Accordingly, attention from Twitter is considered as social interest, attention from news as media and attention from Wikipedia as educational (Torres-Salinas et al., 2022). The consideration of Wikipedia as an educational source can be seen in the fact that it is one of the most used educational resources by students (Selwyn and Gorard, 2016), having a positive impact on their academic performance (Meseguer-Artola et al., 2020), so its mentions can be viewed as a reflection of educational interest. Although each of the social media is approached separately, in all cases we aim not only to identify the different types of audiences who make mentions of these publications, but also several metrics that allow us to characterize the mentions according to three dimensions:

- Local attention: metrics associated with the number of local mentions.
- Mention-engagement: metrics associated with the mention that can reflect its outreach.
- Actor-profile: metrics associated with the actor who made the mention that can reflect the actor's influence and potential community.

At each stage of this proposal, the recommendations made by Thelwall (2020a) are taken into consideration to avoid common problems when dealing with altmetric data to measure social attention. Firstly, each social media source is considered separately to exploit their unique nature, as well as their specific attention, avoiding treating them all identically or recurring to aggregate indicators (Table 1). Secondly, a homogeneous sample of publications is used from the same research field, in a specific period and with authors who belong to the same institution. Thirdly, the altmetric sources chosen are those with the most mentions, with the aim of reducing the problem of zeros. More detailed and specific information can be found in the individual case sections.

**Table 1:** External contextualizing sources used by social media and objective.

**Social media**	**External contextualizing sources**	**Objective**
**Twitter**	Open dataset of Mongeon et al. (2022) Botometer API^*^	Audience identification
	Twitter API	Mention-engagement and actor profile metrics
**News**	Site Traffic API^*^	Audience identification
		Mention-engagement and actor profile metrics
**Wikipedia**	ORES API	Audience identification
	Wikimedia REST API XTools API	Mention-engagement and actor profile metrics

^*^Fee-based application.

## 3. Evaluating social attention through twitter

### 3.1. Twitter metadata retrieval and processing

A total of 12,413 Twitter mentions of Environment/Ecology publications were retrieved from Altmetric.com. Firstly, the Twitter API was consulted to retrieve the metadata of the tweets (language, type of tweet and total favorites and retweets) and the tweeters (followers, friends and total tweets) that posted them. Due to inconsistencies in the Twitter data, after the query the tweet dataset was reduced to 11,952 tweets (96.29%) posted by 7,173 tweeters.

In the next step, the tweeters were classified to differentiate between different types of audiences. The tweeters were matched using the open dataset of Mongeon et al. (2022), which identifies nearly 500,000 researchers' Twitter accounts. As a result, a total of 2,541 researchers (35.42% of the total) were identified in our dataset. Secondly, we queried the API of Botometer v4 (Sayyadiharikandeh et al., 2020), the machine learning tool for identification of bot accounts in Twitter. This predictive model returns a score for each tweeter known as a botscore, which reflects how likely it is that a tweeter is a bot based on its activity patterns and the information it provides. The botscore ranges from 0 to 5, where 0 indicates behavior more associated with a non-automated account and 5 reflects bot-like behavior. The threshold is the most problematic issue, as there is no standard measure and it depends on the researchers (Yang et al., 2022). In this case, due to the distribution of the botscore (see Supplementary Figure 1) a threshold of four was set, thus detecting 289 of the tweeters (4.03%) as bots. The remaining 4,343 tweeters (60.55%) were classified as social audience. It is possible to identify more types of audiences on Twitter, such as journals (Nishikawa-Pacher, 2023), but in this case their presence is so small and in this initial approximation we only consider the three previous ones.

After classifying the tweeters by audience, the tweets published in Spanish were identified as local mentions.^4^ Next, the mention-engagement and actor-profile metrics were obtained. At the mention-engagement level the tweets and retweets were separated, a practice previously employed in altmetric studies to reduce all mentions to those that reflect the most evidence of social attention, i.e., non-retweets (Arroyo-Machado et al., 2021). Secondly, for each tweet the number of retweets and favorites were used as metrics of engagement, both of which are not only widely used but have previously been shown to be useful in this regard (Fang et al., 2022a). It should be noted that both metrics do not apply to retweets, as they reflect the values corresponding to the original tweet that has been retweeted but not to the retweet *per se*. At the actor-profile level, the followers were used as a potential metric of the community reached by their activity, friends as a metric of the community with which they interact and total tweets as the volume of their activity in this social media.

### 3.2. Applying twitter for research evaluation

Table 2 summarizes all these metrics by audience type (bots, science or society) highlighting how each of them has different behavior. Bots have the highest average number of tweets (41,796.96) followed by friends (1,833.9), although they have the fewest average number of followers. Accordingly, despite their intense activity they impact a smaller number of tweeters than social actors, who have the largest community with an average of 4,811.68 followers. Most of the mentions also correspond to this latter group, with 6,562 tweets mentioning 6,595 publications. It is also noteworthy that for both the science and society audiences 66% of their mentions are retweets, although this value drops to 15.53% for bots. The latter are also the least engaged, while science is the most engaged with an average of 2.51 retweets and 7.59 favorites.

**Table 2:** Twitter audience reached by the 982 publications in the Environment/Ecology ESI field published by researchers at the University of Granada.

			**Activity**		**Lang**	**Engagement**		**Tweeter profile**		
**Audience**	**Total actors**	**Papers**	**All Tweets**	**RT**	**Local tweets**	**RT Not retweet**	**Fav. Not retweet**	**Follow**.	**Friends**	**Tweets**
**Total**	7,173	982	11,952	7,576	1,938	1.8	4.75	3,969.8	1,531.32	29,370.79
						0	1	807	744	4,914
Bots	289	378	676	105	35	0.23	0.48	2,236.8	1,833.9	41,796.96
						0	0	332	72	5,745
Science	2,541	650	4,714	3,113	490	2.51	7.59	2,727.97	1,453.01	11,362.4
						1	2	975	849	2,949
Society	4,343	775	6,562	4,358	1,413	1.69	3.8	4,811.68	1,557.01	39,080.25
						0	1	732	709	6,899

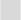
 Mean 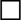 Median.

**Figure 2:**
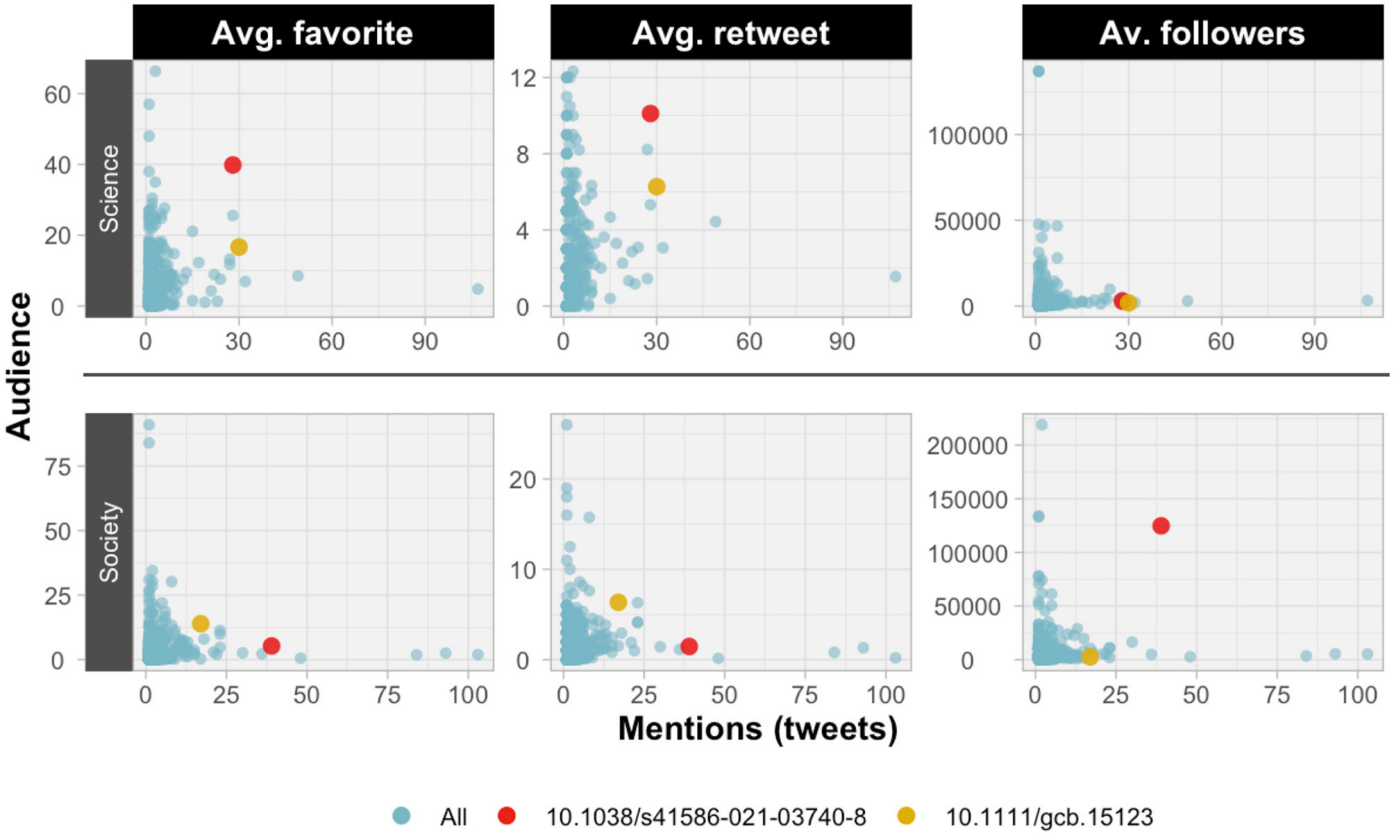
Scatter plot of Environment/Ecology ESI field publications comparing the number of mentions on Twitter and the average indicators of favorites, likes and followers.

The value of these metrics can be seen at publication level, as they provide a better contextualization of the social attention captured through Twitter. Figure 2 shows how, apart from the number of times a paper is mentioned, there are multiple dimensions that give a broader view of social attention and the type of audience it has impacted. The paper by Seibold et al. (2021) has been marked in red and the paper by Lembrechts et al. (2020) in yellow to better exemplify this. In terms of the interest they have aroused in the science audience, both achieved a very similar number of mentions with the latter being slightly higher. However, the average number of times the tweet in which they are mentioned is marked as a favorite or retweeted is much higher in the first case, thus resulting in greater engagement in this community. This interpretation is inverted when looking at the society audience, as it is the second paper that remains behind in mentions but achieves a higher engagement, even though the potential community of the first paper is considerably higher.

**Figure 3:**
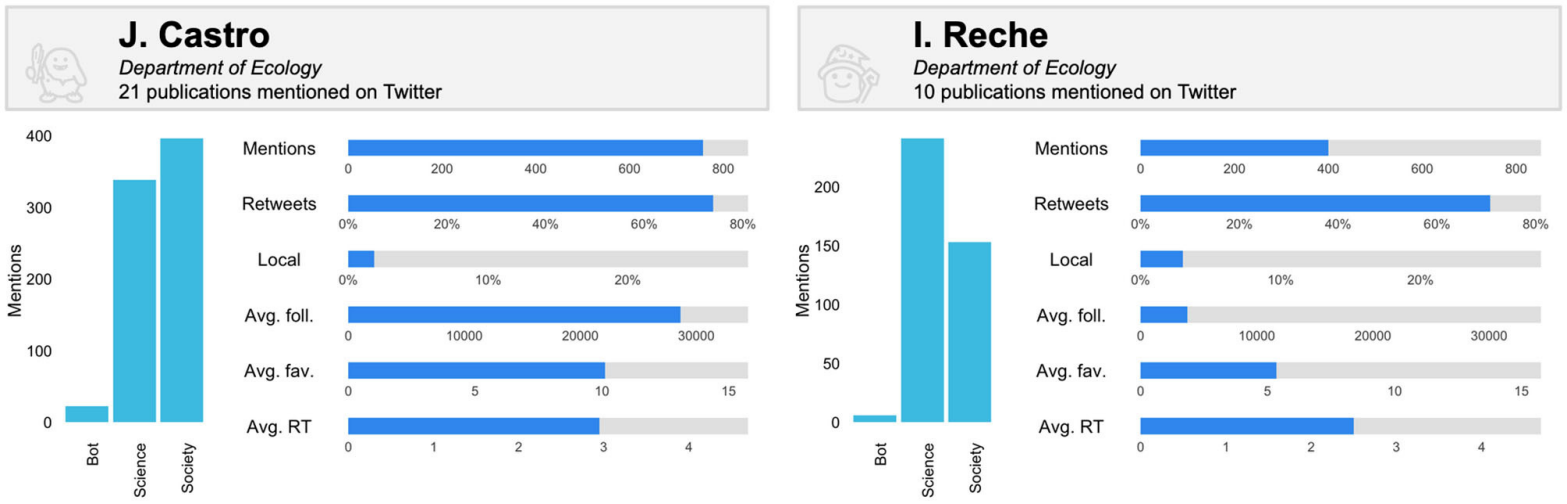
Comparison of audience and social attention captured on Twitter of two authors from the University of Granada who publish in the field of Environment/Ecology (metrics upper limits correspond to the highest value of each metric for authors with five or more papers with mentions on Twitter).

These differences are also evident at author level, revealing different social attention profiles. Figure 3 shows the metrics of two researchers with an unequal number of mentions, the first of them being one of the most mentioned of the group of researchers analyzed. The percentage of retweets and the percentage of tweets in the local language are not very different, although both are slightly higher in the case of Castro. However, the differences become palpable when looking at the audiences. While Castro has a greater social audience, Reche has a greater audience within the scientific community. These differences in audience are reflected in the attention and outreach of the tweets. Firstly, the potential community of Castro considered as the average number of followers of the actors mentioning his publications is much higher, as well as the average number of favorites received by the tweets mentioning his publications. This gap is also appreciable in the average number of retweets, although it is not as striking. Accordingly, apart from their differences in terms of mentions in raw numbers, these two authors have an impact on very different audiences and with equally different engagements.

## 4. Evaluating media attention through news outlets

### 4.1. News outlets metadata retrieval and processing

In the case of Environment/Ecology publications, news mentions are the most extensive after Twitter, with a total of 1,513 mentions retrieved from Altmetric.com. However, it is necessary to highlight a difference with respect to other sources: the actors are completely decentralized. Instead of having a single platform where all the actors move and interact, as for example in the case of tweeters on Twitter, each of the news outlets is an independent website. In fact, the tracking process by Altmetric.com is more biased than with other altmetric sources because it is based on a curated list of news outlets. Accordingly, despite the lack of transparency in the selection of media, Altmetric.com not only offers greater coverage than other aggregators (Ortega, 2018), the accuracy of identification comes before exhaustiveness (Fleerackers et al., 2022), thus achieving highly useful mentions with limited noise. Aside from the strengths and weaknesses of this altmetric source, for metadata retrieval we decided to use the Site Traffic API,^5^ which returns a collection of metrics for a web domain related to its web traffic and classifies them by subject categories.

As was the case with Twitter, the initial number of mentions was reduced after the data was processed. This was due to problems with the Altmetric.com data, as 139 mentions did not include the link to the news story and therefore the Site Traffic API query could not be queried. Similarly, for 14 of the domains it was impossible to retrieve information from the API, so the number of mentions finally analyzed was 1,324 (87.5%) made by 585 different websites. It is also noteworthy that 471 mentions included shortened URLs, a problem that had to be solved before querying the Site Traffic API. Once the data had been retrieved from the API, we made use of the subject category it offers for each website, which is associated with a classification consisting of more than one level. For example, the root category “health” has the subcategories “medicine” and “women's health,” among others. That is why in our case, given the volume of different subcategories in the study, the decision was made to reduce the subcategories to their 16 root categories. Furthermore, to make an even broader division these root categories were classified into two blocks: the root category “news and media” and the rest (e.g., arts and entertainment, finance, health…), thus making it possible to consider the former as mainstream media (e.g., theguardian.com or cnn.com) and the latter as specialist media (e.g., weather.com or techtimes.com).

In relation to the API metadata used, news outlets for which Spain was the country with the highest volume of traffic were identified as local. For the engagement metrics, the raw number of visits, the average number of pages of the website accessed by visitors and the average number of seconds spent on the website were selected. To characterize the news outlets, we used their position in the global ranking and their bounce rate, the latter being a well-known measure in the SEO and web industry that indicates the percentage of visitors who leave the website without clicking on it, indicating a lack of interactivity. Both metrics should be read with caution, as the lower the value, the higher the relevance.

### 4.2. Applying news outlets for research evaluation

Table 3 summarizes all metrics by audience type. Most of the websites belong to mainstream media, with 351 news outlets responsible for 710 of the mentions (53%), while specialist media account for 234 news outlets including 614 mentions (46%). While most of the mentions of local news outlets are from mainstream media, the science and education specialist media have the highest percentage of local mentions (21%). In terms of engagement metrics for mentions and characterization of news outlets, the mainstream media are the most prominent. However, this comparison varies when looking at certain categories, such as the 20 news outlets for “computers, electronics and technology” and the 18 for “finance,” which for some of the metrics have the best values. All these differences show how the media attention is highly different depending on the news outlets.

**Table 3:** News outlets audience reached by the 164 publications in the Environment/Ecology ESI field published by researchers at the University of Granada.

				**Activity**	**Lang**	**Engagement**			**News outlets profile**	
**Audience**	**Webs**	**Local**	**Papers**	**Mentions**	**Local ment**.	**Visits (million)**	**Pages**	**Time (s)**	**Rank**	**Bounce**
**Total**	585	56	164	1,324	208	24.82	2.01	128.18	774,833.72	0.65
						1.06	1.79	83.93	67,251	0.67
Mainstream media	351	41	108	710	130	33.92	2.1	139.93	760,004.6	0.63
						1.42	1.91	97.99	46,887.5	0.64
Specialist media**↓**	234	15	121	614	78	11.17	1.88	110.55	797,285.27	0.67
(**↓** root categories)						0.67	1.66	69.96	101,414.5	0.7
**Arts and entertainment** specialist media	79	3	22	144	9	5.73	1.88	124.76	498,850.67	0.65
						0.61	1.72	80.24	123,812	0.68
**Science and education** specialist media	63	4	91	255	54	21.1	1.79	73.32	941,680.14	0.72
						0.32	1.56	56.87	208,413	0.74
**Health** specialist media	34	4	34	80	7	12.55	1.73	68.27	348,170.66	0.67
						1.72	1.63	63.63	41,227.5	0.72
**Computers elect. and tec**. specialist media	20	1	20	58	1	6.63	2.2	175.27	131,899.47	0.61
						3.71	1.78	94.83	34,386	0.68
**Finance** specialist media	18	1	11	26	1	9.62	2.24	217.62	1,259,228.29	0.65
						2.99	1.82	63.09	25,206.5	0.67
**Other** (10 categories) specialist media	20	2	35	51	6	4.92	1.76	82.52	2,821,274.12	0.67
						0.34	1.8	72.57	187,842	0.69

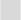
 Mean 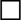 Median.

**Figure 4:**
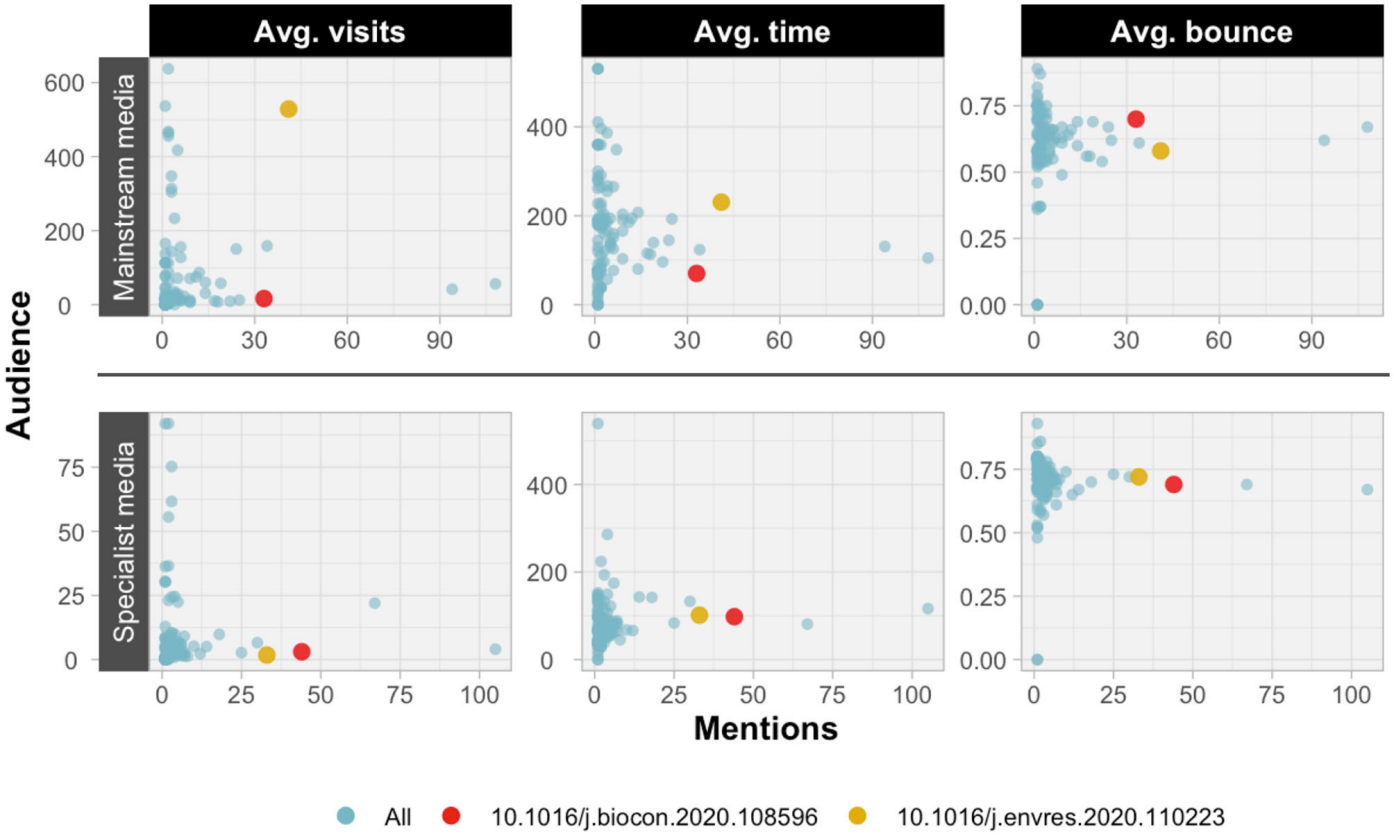
Scatter plot of Environment/Ecology ESI field publications comparing the number of mentions on news outlets and the average indicators of visits, time and bounce.

This multidimensionality in media attention is highlighted in Figure 4, which compares the news mentions of publications with the average bounce rate, visits and visit time of the news outlets that make such mentions, further differentiating between the two main audiences. Two papers have been highlighted to exemplify such differences, with the paper by Carlson et al. (2020) marked in red and the paper by Rodríguez-Barranco et al. (2021) in yellow. In both cases the mention counts are very similar, the former with a slight advantage in specialist media and the latter in mainstream media. However, while the latter is mentioned in mainstream media with a substantially higher number of visits and average visit time, in specialist media the differences are not very noticeable. Something similar occurs with the bounce rate. Accordingly, while both are mentioned in specialist media with very similar relevance, the mainstream media that pay attention to both are completely different, with those that mention the paper by Rodríguez-Barranco et al. (2021) being among the most relevant.

**Figure 5:**
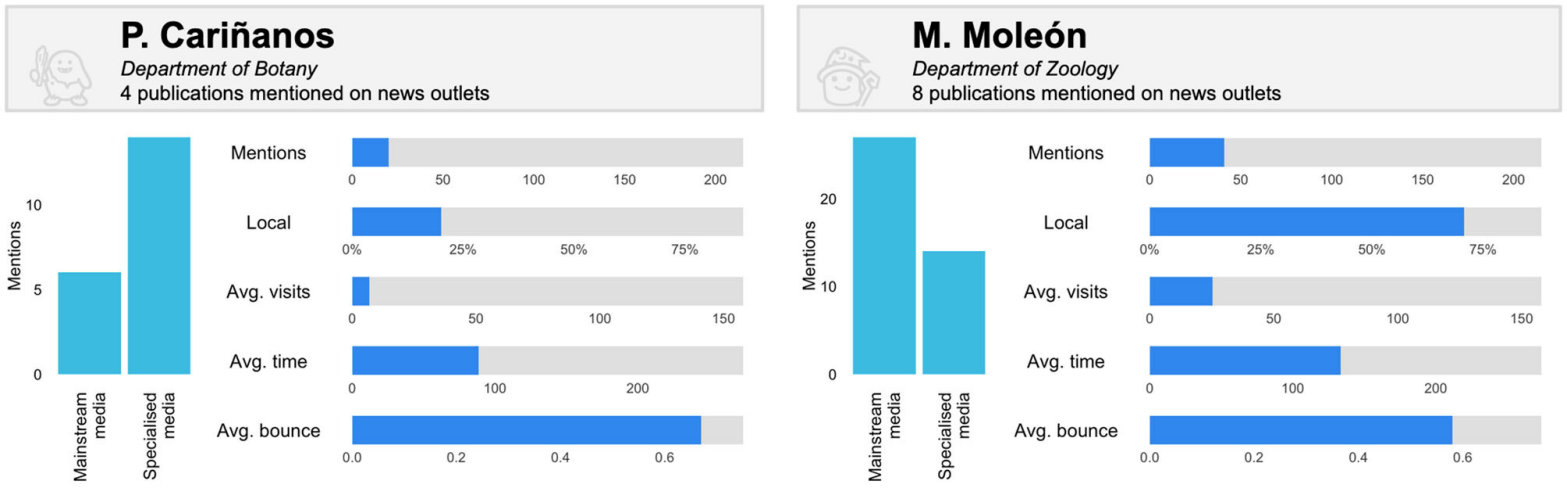
Comparison of audience and media attention captured on news outlets for two authors from the University of Granada publishing on Environment/Ecology (metrics upper limits correspond to the highest value of each metric for authors with three or more papers with mentions on news outlets).

At author level, these differences are even more noticeable and comprehensible. Figure 5 includes comparisons of two authors with a similar number of mentions, with Moleón having a slightly higher value. The audiences of the two authors are different; Cariñanos attracts more attention from the specialist media than the mainstream media, the exact opposite of Moleón. Similarly, the publications by Moleón have noticeably more local media attention than those of Cariñanos. This is also reflected in the average number of visits to the news outlets, which is higher in the case of Moleón, although the bounce rate is slightly lower. It can thus be concluded that while Cariñanos has more of a specialist and international media attention profile, Moleón attracts more attention from local and more visible news outlets.

## 5. Evaluating educational attention through wikipedia

### 5.1. Wikipedia metadata retrieval and processing

In the case of Wikipedia, the mentions retrieved from Altmetric.com are the least numerous, with a total of 79 mentions. However, Wikipedia data is the most accessible of the three media studied as it is completely open and has multiple access points, ranging from data dumps to APIs (Arroyo-Machado et al., 2022). Similarly, the range of metrics that can be used to characterize the attention and engagement of mentions on Wikipedia is diverse. The Wikimedia REST API, XTools API and ORES API have all been used to contextualize these mentions.

As in the previous cases, during the metadata retrieval process five mentions were eliminated as their pages were no longer available. To identify the audiences we used ORES, an artificial intelligence application which detects vandalism in edits of pages and can also predict a topic for the page according to a taxonomy. In this case, as was done for the news, we selected only the top level of this classification (Culture, Geography, History and Society and STEM). Moreover, since this option is not currently available in all languages, in those cases where a topic category could not be predicted because of the language, the English version of the page was used to identify the topic. Since not all pages have an English Wikipedia version, the language could not be identified for six of the pages. Meanwhile, for the identification of local mentions, all those coming from pages relating to edits of languages spoken in Spain were marked as such, in this case the Spanish and Catalan Wikipedia. Regarding the engagement metrics, the page views and number of edits of the Wikipedia pages that make the mentions were used. For the characterization of the pages, the number of words contained on the page, the number of other languages in which the page is translated and the total number of references made were used. These metrics have already demonstrated their potential in the past to capture attention and characterize the profile of Wikipedia pages (Mittermeier et al., 2021).

### 5.2. Applying wikipedia for research evaluation

Table 4 summarizes all metrics by audience type. Among the different audiences, the most prominent is STEM with a total of 44 pages (69%) and 51 mentions (69%). When analyzing the engagement of the different types of audiences, it is precisely this same audience that has the highest average number of page views, although the pages with the highest average number of edits are those of History and Society. This suggests that while the former attract the most interest, it is the latter that are the most active in terms of content. This is reinforced by the fact that the History and Society pages are the ones with the largest number of words, translations and references, indicating that they are pages with a high level of content and development of the topic they deal with. This profile is similar to that of the Culture pages, although they are less international. In general terms, the existence of different audiences with different profiles is once again appreciable.

**Table 4:** Wikipedia audience reached by the 44 publications in the Environment/Ecology ESI field published by researchers at the University of Granada.

				**Activity**		**Lang**	**Engagement**		**Page profile**		
**Audience**	**Pages**	**Langs**	**Papers**	**Ment**.	**Users**	**Local ment**.	**Views**	**Edits**	**Words**	**Transl**.	**Ref**.
**Total**	64	17	44	74	59	10	382,792.75	453.86	2,692.94	21.33	116.41
							21,069.5	68	902.5	9	38
Culture	7	5	3	7	6	2	107,631.43	183.57	4,751.29	12.86	203.43
							17,505	49	932	9	37
Geography	2	2	2	2	2	0	24,087.5	67	1,345.5	21	78.5
							24,087.5	67	1,345.5	21	78.5
History and Society	5	3	6	8	6	0	862,605	1,444.4	9,412	61.6	335
							95,371	310	11,306	45	319
STEM	44	14	35	51	40	6	439,161.86	461.75	1,852.68	20.84	91.59
							27,940.5	79.5	779	10.5	33.5
Unknown	6	3	5	6	6	2	10,165.67	14.83	1,303.33	1.33	27.33
							1,121.5	14.5	607	1	11

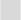
 Mean 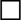 Median.

**Figure 6:**
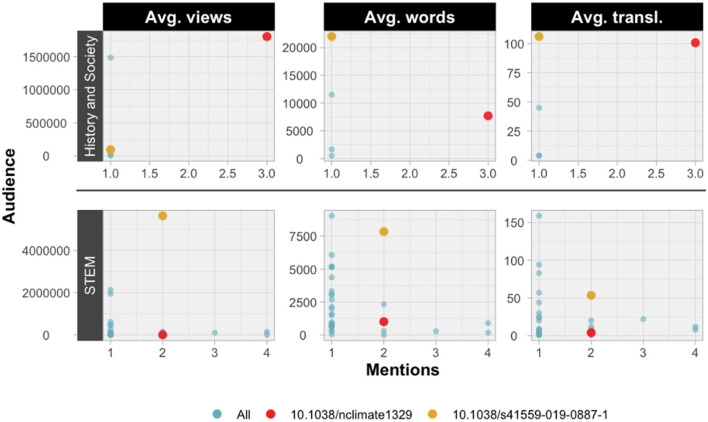
Scatter plot of Environment/Ecology ESI field publications comparing the number of mentions on Wikipedia and the average indicators of views, words and translations.

The different perspectives of the proposed metrics for the contextualization of educational attention on Wikipedia and its main audiences are highlighted in Figure 6. Two publications have been highlighted to exemplify these differences, with the paper by Gottfried et al. (2012) marked in red and the letter by Cortés-Sánchez et al. (2019) in yellow. Both are mentioned by the two main audiences (History and Society, and STEM). The paper has more mentions in History and Society pages, which, despite not having a very high average number of words, are among the main ones in terms of translations and page views. Meanwhile, the letter receives the same number of mentions in STEM pages as the paper but it is mentioned in pages with a higher number of words, translations and, above all, page views. This shows that the attention received by both audiences is unequal, with greater relevance in the History and Society pages that mention the paper, as well as in the STEM pages that mention the letter.

**Figure 7:**
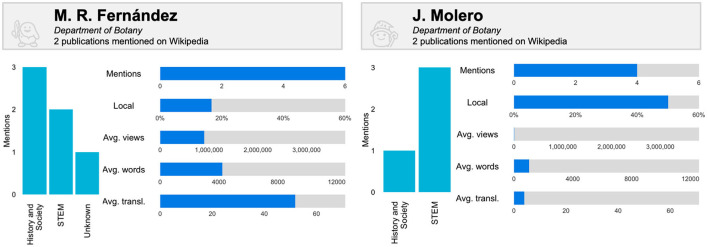
Comparison of the audience and educational attention captured on Wikipedia of two authors from the University of Granada who publish on Environment/Ecology (metrics upper limits correspond to the highest value of each metric for authors with 2 or more papers with mentions on Wikipedia).

Finally, two authors are compared again to demonstrate the potential of these metrics (Figure 7). Each of them has a similar number of mentions of their publications in Wikipedia, but their contextualization reveals relevant insights into this attention. Firstly, the audience of Fernández is mostly History and Society, with most mentions coming from non-local pages. The opposite holds true for Molero, whose mainly STEM audience has more local attention. Such international and local interest may be influential in terms of page views, development of content and translations, as Fernández is significantly superior in all these aspects.

## 6. Discussion

This paper provides a framework for applying altmetrics in a meaningful way in the context of university evaluation for decision-making. Twitter has been used for the study of social attention, news for media and Wikipedia for education. Each social media source has been approached independently, but seeking in all cases to identify the different audiences, local attention, engagement metrics and characterization of the actors responsible for these mentions. One of the most relevant contributions is that it illustrates in a practical way how indicators can be combined with unique sources of information to allow contextualization of attention. More specifically, it shows how tools not commonly used in altmetric studies such as Botometer and Site Traffic API can qualify and give meaning to metrics. In this sense, another valuable contribution is the link made between altmetrics and the practical postulates or principles of evaluative bibliometrics. This places the paper in line with the contributions of Henk F. Moed, who indicated the need for large informetric datasets to offer more robust metrics. He also mentioned the need for extra-informetric factors to determine the wider social context (Moed, 2017). We believe that this work sheds light on how context metrics can also be applied to altmetrics.

This study is also unique in terms of its applied aspects, as it also follows the recommendations made by Thelwall (2020a) to avoid the well-known problems and overcome the main limitations when studying social attention. That is why each social media channel has been individually addressed and explored to take into account their strengths and weaknesses. This approach is diametrically opposed to the generation of aggregate metrics, which can provide an initial approximation of general social attention but also ignore many of the dimensions of the social media involved by establishing random criteria for their aggregation (Gumpenberger et al., 2016). Similarly, we also avoid evaluative malpractice by considering that what these metrics reflect is attention, contextualizing that attention, and ultimately providing useful and easily interpretable tools (Moed et al., 1985). Concerning these practical tools, our proposal advocates the generation of publication-level and author-level dashboards that offer a complete overview of the attention to be measured, clearly separating the different instances of social attention and always considering the research field in which they are evaluated. This is a point to consider in the development of applications that aim to integrate altmetrics into researcher portfolios, an unexploited domain with a limited number of proposals, one of the more noteworthy ones being ImpactStory (Priem and Piwowar, 2012).

Having demonstrated the potential and usefulness of altmetrics in the field of scientific evaluation, the proposed framework could be applied in evaluative contexts for decision-making by institutions and evaluation agencies. Firstly, continuing with the case study used in this paper, there are several possible scenarios for its potential use at institutional level at the University of Granada. It could be used by the Social Council of the University of Granada,^6^ a governing body that represents the interests of society and the relationship between the university and its surrounding environment. This university body could use altmetrics to evaluate the social attention and visibility of its researchers when awarding prizes and grants. Similarly, the International Research Projects Office (OFPI),^7^ the unit for advice, dissemination, promotion and management of international research projects at the University of Granada, could use altmetrics to evaluate the social attention of projects and prepare monitoring reports. Secondly, in terms of evaluation agencies the ANECA (National Agency for Quality Assessment and Accreditation of Spain) should be highlighted. Among its accreditations is the 6 Year Transfer,^8^ which recognizes the transfer of research activity to social agents and companies over a 6 year period. This accreditation provides an ideal framework for the use of altmetrics to facilitate the evaluation of applications.

This proposal and the related research are not free of limitations. Firstly, the well-known and inherent limitations of altmetrics stand out, especially the skewed distribution of data by social media. Secondly, news mentions may be biased, as Altmetric.com uses a curated list of news outlets for tracking. Thirdly, machine learning techniques and datasets derived from their application have been used to detect audiences on Twitter and Wikipedia, meaning that some actors may not be accurately identified. Fourthly, the mention-engagement and actor-profile metrics are as of February 2023, so they may fail to contextualize the impact at the exact time the mention was made. Fifthly, while this methodology is fully reproducible, both the altmetrics data used and some of the complementary sources used are not open, which can be a barrier to large-scale implementation, especially in the case of Twitter and news outlets. Finally, this proposal only considers a small subset of social attention metrics concerning three dimensions (local attention, mention-engagement and actor-profile), but it is possible to consider several more, such as self-dissemination, which is of particular interest in the case of Twitter (Ferreira et al., 2021).

We wish to conclude this paper by calling for the use of altmetrics in evaluative contexts. Although these indicators are a substantial improvement to bean counting, they must be used in addition to peer reviews as bibliometric evaluations have done since the 1980s, an approach that the European Union also encourages in its recommendations (European Commission Directorate-General for Research Innovation et al., 2017). It is time to move away from talking about alternative metrics (Buschman and Michalek, 2013) and incorporate them into the evaluation context by offering precise and practical solutions.

## Data availability statement

The original contributions presented in the study are included in the article/Supplementary material, further inquiries can be directed to the corresponding authors. All the Python and R scripts with data processing are available at the following GitHub repository: https://doi.org/10.5281/zenodo.7740950.

## Author contributions

Both authors have made a substantial contribution to the conception, design, analysis and drafting of the work, and they approved the final manuscript.

## Footnotes

^1^The terms social attention and scientific impact are often used indifferently in the literature. But it is more appropriate to refer to attention as it denotes broad public interest while impact carries more transformative connotations (Sugimoto, 2015).

^2^https://ranking.influscience.eu/ (accessed February 1, 2023).

^3^The percentage of publications with at least one altmetric mention is not far off the results of other studies (Arroyo-Machado et al., 2021) and is strongly influenced by the research field (Torres-Salinas et al., 2023).

^4^Because of the limitations in the location data of the tweeters' profiles (it is not provided by all tweeters and is a non-standardized field), the tweet language has been used as a proxy for the identification of local tweets.

^5^https://zylalabs.com/api-marketplace/data/site+traffic+api/29 (accessed February 1, 2023).

^6^ https://www.ugr.es/en/about/organization/positions/social-council

^7^ https://ofpi.ugr.es/

^8^ https://www.aneca.es/en/web/guest/six-year-transfer

## Supplementary material

SUPPLEMENTARY FIGURE 1 Botscore distribution of tweeters that mention Environment/Ecology ESI field publications authored by researchers at the University of Granada. The dashed line indicates the threshold of 4.

## References

[B1] Arroyo-Machado W. Torres-Salinas D. Costas R. (2022). Wikinformetrics: construction and description of an open Wikipedia knowledge graph data set for informetric purposes. Quant. Sci. Stud. 3, 931–952. 10.1162/qss_a_00226

[B2] Arroyo-Machado W. Torres-Salinas D. Herrera-Viedma E. Romero-Frías E. (2020). Science through Wikipedia: a novel representation of open knowledge through co-citation networks. PLoS ONE 15:e0228713. 10.1371/journal.pone.0228713 32040488 PMC7010282

[B3] Arroyo-Machado W. Torres-Salinas D. Robinson-Garcia N. (2021). Identifying and characterizing social media communities: a socio-semantic network approach to altmetrics. Scientometrics 126, 9267–9289. 10.1007/s11192-021-04167-8 34658460 PMC8507359

[B4] Bornmann L. (2014). Do altmetrics point to the broader impact of research? an overview of benefits and disadvantages of altmetrics. J. Informetr. 8, 895–903. 10.1016/j.joi.2014.09.005

[B5] Bornmann L. Haunschild R. Adams J. (2019). Do altmetrics assess societal impact in a comparable way to case studies? an empirical test of the convergent validity of altmetrics based on data from the UK research excellence framework (REF). J. Informetr. 13, 325–340. 10.1016/j.joi.2019.01.008

[B6] Buschman M. Michalek A. (2013). Are alternative metrics still alternative? Bull. Am. Soc. Inf. Sci. Technol. 39, 35–39. 10.1002/bult.2013.1720390411

[B7] Carlson C. J. Hopkins S. Bell K. C. Doña J. Godfrey S. S. Kwak M. L.. (2020). A global parasite conservation plan. Biol. Conserv. 250:108596. 10.1016/j.biocon.2020.108596

[B8] Caron E. van Eck N. -J. (2014). “Large scale author name disambiguation using rule-based scoring and clustering: International conference on science and technology indicators,” in Proceedings of the Science and Technology Indicators Conference 2014, eds E. Noyons (Leiden: Universiteit Leiden), 79–86. Available online at: http://sti2014.cwts.nl (accessed April 17, 2022).

[B9] Cortés-Sánchez M. Jiménez-Espejo F. J. Simón-Vallejo M. D. Stringer C. Lozano Francisco M. C. García-Alix A.. (2019). Reply to ‘dating on its own cannot resolve hominin occupation patterns' and ‘no reliable evidence for a very early Aurignacian in Southern Iberia.' Nat. Ecol. Evol. 3, 714–715. 10.1038/s41559-019-0887-1 30988498

[B10] Costas R. de Rijcke S. Marres N. (2020). “Heterogeneous couplings”: operationalizing network perspectives to study science-society interactions through social media metrics. J. Assoc. Inf. Sci. Technol. 72, 595–610. 10.1002/asi.24427

[B11] Costas R. Zahedi Z. Wouters P. (2015). Do “altmetrics” correlate with citations? extensive comparison of altmetric indicators with citations from a multidisciplinary perspective. J. Assoc. Inf. Sci. Technol. 66, 2003–2019. 10.1002/asi.23309

[B12] Díaz-Faes A. A. Bowman T. D. Costas R. (2019). Towards a second generation of ‘social media metrics': characterizing Twitter communities of attention around science. PLoS ONE 14:e0216408. 10.1371/journal.pone.0216408 31116783 PMC6530891

[B13] European Commission Directorate-General for Research Innovation Peters, I. Frodeman R. Wilsdon J. Bar-Ilan J. Elisabeth L.. (2017). Next-Generation Metrics: Responsible Metrics and Evaluation for Open Science. Brussels: Publications Office

[B14] European Commission Directorate-General for Research Innovation Schomberg R. Britt Holbrook J. Oancea A. Kamerlin S. Ismael R.. (2019). Indicator Frameworks for Fostering Open Knowledge Practices in Science and Scholarship. Brussels: Publications Office

[B15] Fang Z. Costas R. Tian W. Wang X. Wouters P. (2020). An extensive analysis of the presence of altmetric data for Web of Science publications across subject fields and research topics. Scientometrics 124, 2519–2549. 10.1007/s11192-020-03564-9 32836523 PMC7297939

[B16] Fang Z. Costas R. Tian W. Wang X. Wouters P. (2021). How is science clicked on Twitter? click metrics for bitly short links to scientific publications. J. Assoc. Inf. Sci. Technol. 72, 918–932. 10.1002/asi.24458

[B17] Fang Z. Costas R. Wouters P. (2022a). User engagement with scholarly tweets of scientific papers: a large-scale and cross-disciplinary analysis. Scientometrics 127, 4523–4546. 10.1007/s11192-022-04468-6

[B18] Fang Z. Dudek J. Costas R. (2022b). Facing the volatility of tweets in altmetric research. J. Assoc. Inf. Sci. Technol. 73, 1192–1195. 10.1002/asi.24624

[B19] Ferreira M. R. Mongeon P. Costas R. (2021). Large-scale comparison of authorship, citations and tweets of web of science authors. J. Altmetrics 4, 1. 10.29024/joa.38

[B20] Fleerackers A. Nehring L. Maggio L. A. Enkhbayar A. Moorhead L. Alperin J. P. (2022). Identifying science in the news: an assessment of the precision and recall of Altmetric.com news mention data. Scientometrics 127, 6109–6123. 10.1007/s11192-022-04510-7 36212767 PMC9526208

[B21] Gottfried M. Pauli H. Futschik A. Akhalkatsi M. Barančok P. Benito Alonso J. L.. (2012). Continent-wide response of mountain vegetation to climate change. Nat. Clim. Change 2, 111–115. 10.1038/nclimate1329

[B22] Gumpenberger C. Glänzel W. Gorraiz J. (2016). The ecstasy and the agony of the altmetric score. Scientometrics 108, 977–982. 10.1007/s11192-016-1991-5

[B23] InfluScience (2022). Sobre InfluScience. Plataforma Influscience. Available online at: https://ranking.influscience.eu/sobre-influscience/ (accessed March 6, 2023).

[B24] Karmakar M. Banshal S. K. Singh V. K. (2021). A large-scale comparison of coverage and mentions captured by the two altmetric aggregators: Altmetric.com and PlumX. Scientometrics 126, 4465–4489. 10.1007/s11192-021-03941-y

[B25] Lembrechts J. J. Aalto J. Ashcroft M. B. De Frenne P. Kopecký M. Lenoir J.. (2020). SoilTemp: a global database of near-surface temperature. Glob. Change Biol. 26, 6616–6629. 10.1111/gcb.15123 32311220

[B26] Lewoniewski W. Wecel K. Abramowicz W. (2017). “Analysis of references across Wikipedia languages,” in Information and Software Technologies, eds. R. Damaševičius and V. Mikašyte (Cham: Springer International Publishing), 561–573. 10.1007/978-3-319-67642-5_47

[B27] Maggio L. A. Willinsky J. M. Steinberg R. M. Mietchen D. Wass J. L. Dong T. (2017). Wikipedia as a gateway to biomedical research: the relative distribution and use of citations in the english Wikipedia. PLoS ONE 12:e0190046. 10.1371/journal.pone.0190046 29267345 PMC5739466

[B28] Meseguer-Artola A. Rodríguez-Ardura I. Ammetller G. Rimbau-Gilabert E. (2020). Academic impact and perceived value of Wikipedia as a primary learning resource in higher education. Profesional de la información. 29, e290329. 10.3145/epi.2020.may.29

[B29] Mittermeier J. C. Correia R. Grenyer R. Toivonen T. Roll U. (2021). Using Wikipedia to measure public interest in biodiversity and conservation. Conserv. Biol. 35, 412–423. 10.1111/cobi.13702 33749051

[B30] Moed H. F. (2017). Applied Evaluative Informetrics. Cham: Springer International Publishing. 10.1007/978-3-319-60522-7

[B31] Moed H. F. Burger W. J. M. Frankfort J. G. Van Raan A. F. J. (1985). The use of bibliometric data for the measurement of university research performance. Res. Policy 14, 131–149. 10.1016/0048-7333(85)90012-5

[B32] Mongeon P. Bowman T. D. Costas R. (2022). An Open Dataset of Scholars on Twitter. 10.1162/qss_a_00250

[B33] National Information Standards Organization (2016). NISO RP-25-2016 Outputs of the NISO Alternative Assessment Metrics Project. NISO website. Baltimore, MD. Available online at: https://www.niso.org/publications/rp-25-2016-altmetrics (accessed March 6, 2023).

[B34] Nielsen F. A. (2007). Scientific Citations in Wikipedia. First *Monday*, 12. 10.5210/fm.v12i8.1997

[B35] Nishikawa-Pacher A. (2023). The Twitter accounts of scientific journals: a dataset. Insights UKSG J. 36, 1. 10.1629/uksg.59334653016

[B36] Ortega J. L. (2018). Reliability and accuracy of altmetric providers: a comparison among Altmetric.com, PlumX and crossref event data. Scientometrics 116, 2123–2138. 10.1007/s11192-018-2838-z

[B37] Ortega J. L. (2021). How do media mention research papers? structural analysis of blogs and news networks using citation coupling. J. Informetr. 15:101175. 10.1016/j.joi.2021.101175

[B38] Priem J. Piwowar H. (2012). The Launch of ImpactStory: Using Altmetrics to Tell Data-Driven Stories. Impact of Social Sciences. Available online at: https://blogs.lse.ac.uk/impactofsocialsciences/2012/09/25/the-launch-of-impactstor/ (accessed May 14, 2023).

[B39] Priem J. Piwowar H. A. Hemminger B. M. (2012). Altmetrics in the Wild: Using Social Media to Explore Scholarly Impact. ArXiv.org. Available online at: https://arxiv.org/abs/1203.4745

[B40] Priem J. Taraborelli D. Groth P. Neylon C. (2010). Altmetrics: A manifesto. Altmetrics. Available online at: http://altmetrics.org/manifesto/

[B41] Ràfols I. (2019). SandT indicators in the wild: contextualization and participation for responsible metrics. Res. Eval. 28, 7–22. 10.1093/reseval/rvy030

[B42] Robinson-Garcia N. van Leeuwen T. N. Ràfols I. (2018). Using altmetrics for contextualised mapping of societal impact: from hits to networks. Sci. Public Policy 45, 815–826. 10.1093/scipol/scy024

[B43] Rodríguez-Barranco M. Rivas-García L. Quiles J. L. Redondo-Sánchez D. Aranda-Ramírez P. Llopis-González J.. (2021). The spread of SARS-CoV-2 in Spain: hygiene habits, sociodemographic profile, mobility patterns and comorbidities. Environ. Res. 192:110223. 10.1016/j.envres.2020.110223 32971081 PMC7505892

[B44] Sayyadiharikandeh M. Varol O. Yang K.-C. Flammini A. Menczer F. (2020). “Detection of novel social bots by ensembles of specialized classifiers,” in Proceedings of the 29th ACM International Conference on Information and Knowledge Management CIKM'20 (New York, NY, USA: Association for Computing Machinery), 2725–2732. 10.1145/3340531.3412698

[B45] Schalkwyk F. Dudek J. Costas R. (2020). Communities of shared interests and cognitive bridges: the case of the anti-vaccination movement on Twitter. Scientometrics 152, 1499–1516. 10.1007/s11192-020-03551-0

[B46] Seibold S. Rammer W. Hothorn T. Seidl R. Ulyshen M. D. Lorz J.. (2021). The contribution of insects to global forest deadwood decomposition. Nature 597, 77–81. 10.1038/s41586-021-03740-8 34471275

[B47] Selwyn N. Gorard S. (2016). Students' use of Wikipedia as an academic resource—patterns of use and perceptions of usefulness. Internet Higher Educ. 28, 28–34. 10.1016/j.iheduc.2015.08.004

[B48] Sugimoto C. (2015). “*Attention is not Impact” and Other Challenges for Altmetrics. The Wiley Network*. Available online at: https://www.wiley.com/en-us/network/publishing/research-publishing/promoting-your-article/attention-is-not-impact-and-other-challenges-for-altmetrics (accessed May 12, 2023).

[B49] Sugimoto C. R. Work S. Larivière V. Haustein S. (2017). Scholarly use of social media and altmetrics: a review of the literature. J. Assoc. Inf. Sci. Technol. 68, 2037–2062. 10.1002/asi.23833

[B50] Thelwall M. (2018a). Early mendeley readers correlate with later citation counts. Scientometrics 115, 1231–1240. 10.1007/s11192-018-2715-9

[B51] Thelwall M. (2018b). “Using altmetrics to support research evaluation,” *in Altmetrics for Research Outputs Measurement and Scholarly Information Management*, eds M. Erdt, A. Sesagiri Raamkumar, E. Rasmussen and Y.-L. Theng (Singapore: Springer Singapore), 11–28. 10.1007/978-981-13-1053-9_2

[B52] Thelwall M. (2020a). Measuring societal impacts of research with altmetrics? common problems and mistakes. J. Econ. Surv. 35. 10.1111/joes.12381

[B53] Thelwall M. (2020b). The Pros and Cons of the Use of Altmetrics in Research Assessment. Scholarly Assessment Reports. p. 2. 10.29024/sar.10

[B54] Thelwall M. Haustein S. Larivière V. Sugimoto C. R. (2013). Do altmetrics work? twitter and ten other social web services. PLoS ONE 8:e64841. 10.1371/journal.pone.0064841 23724101 PMC3665624

[B55] Torres-Salinas D. Clavijo Á. C. Contreras E. J. (2013). Altmetrics: nuevos indicadores para la comunicación científica en la Web 2.0 - altmetrics: new indicators for scientific communication in web 2.0. Rev. Comun. 21, 53–60. 10.3916/C41-2013-05

[B56] Torres-Salinas D. Docampo D. Arroyo-Machado W. Robinson-Garcia N. (2023). The Many Publics of Science: Using Altmetrics to Identify Common Communication Channels by Scientific field. Available online at: http://arxiv.org/abs/2304.05157 (accessed April 21, 2023).

[B57] Torres-Salinas D. Robinson-García N. Arroyo-Machado W. (2022). Coverage and distribution of altmetric mentions in Spain: a cross-country comparison in 22 research fields. Profesional de la información. 31, e310220. 10.3145/epi.2022.mar.20

[B58] Torres-Salinas D. Robinson-Garcia N. Jiménez-Contreras E. (2016). Can We Use Altmetrics At the Institutional Level? A Case Study Analysing the Coverage by Research Areas of Four Spanish Universities. in (Valencia, Spain). Available online at: http://arxiv.org/abs/1606.00232 (accessed March 15 2023).

[B59] Yang K. -C. Ferrara E. Menczer F. (2022). Botometer 101: social bot practicum for computational social scientists. J. Comput. Soc. Sci. 5, 1511–1528. 10.1007/s42001-022-00177-5 36035522 PMC9391657

[B60] Yu H. Li L. Cao X. Chen T. (2022). Exploring country's preference over news mentions to academic papers. J. Informetr. 16:101347. 10.1016/j.joi.2022.101347

[B61] Zahedi Z. Costas R. (2018). General discussion of data quality challenges in social media metrics: extensive comparison of four major altmetric data aggregators. PLoS ONE 13:e0197326. 10.1371/journal.pone.0197326 29772003 PMC5957428

