# Corrigendum: Evaluative altmetrics: is there evidence for its application to research evaluation?

**DOI:** 10.3389/frma.2023.1295959

**Published:** 2023-10-17

**Authors:** Wenceslao Arroyo-Machado, Daniel Torres-Salinas

**Affiliations:** Department of Information and Communication Sciences, University of Granada, Granada, Spain

**Keywords:** altmetrics, social media metrics, research evaluation, evaluative altmetrics, Twitter, news, Wikipedia

In the published article, there was an error in the Funding statement. The projects indicated (PID2019-109127RB-I00/SRA/10.13039/501100011033 and A-SEJ-638-UGR20) are not those associated with this research and which will be responsible for funding. The correct Funding statement appears below.

