# Corrigendum: Corrigendum: Evaluative altmetrics: is there evidence for its application to research evaluation?

**DOI:** 10.3389/frma.2023.1331874

**Published:** 2023-12-01

**Authors:** Wenceslao Arroyo-Machado, Daniel Torres-Salinas

**Affiliations:** Department of Information and Communication Sciences, University of Granada, Granada, Spain

**Keywords:** altmetrics, social media metrics, research evaluation, evaluative altmetrics, Twitter, news, Wikipedia

The previously corrected reference to the project is incomplete. The correct Funding statement appears below.

