# Supplementary figures and images for: Evaluative altmetrics: is there evidence for its application to research evaluation?
Source: Front Res Metr Anal. 2023 Jul 25;8:1188131. doi: 10.3389/frma.2023.1188131 (PMC10407088; doi:10.3389/frma.2023.1188131)

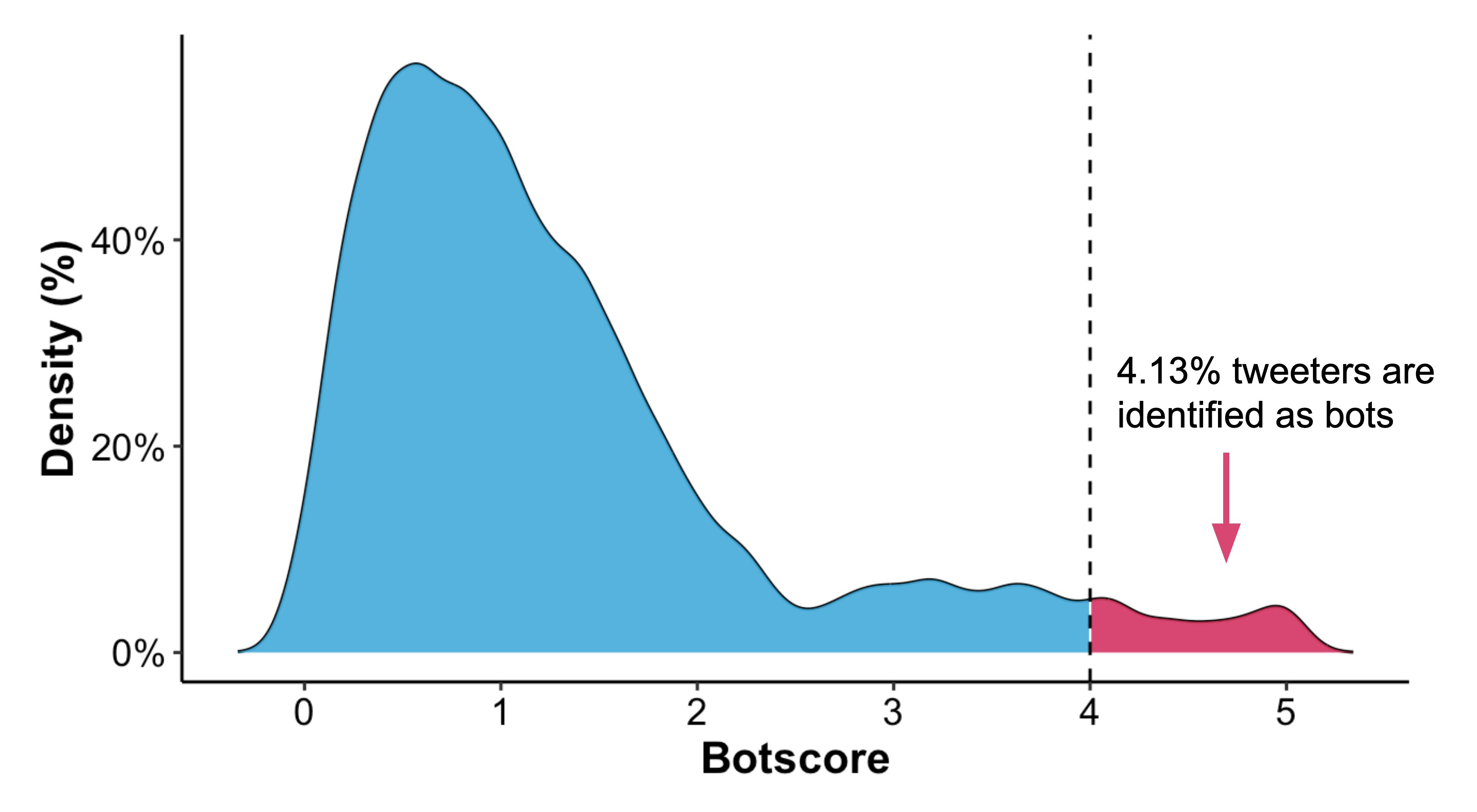

Supplement: SUPPLEMENTARY FIGURE 1 — Botscore distribution of tweeters that mention Environment/Ecology ESI field publications authored by researchers at the University of Granada. The dashed line indicates the threshold of 4. [file Image_1.JPEG]
